# Household heating associated with disability in activities of daily living among Chinese middle-aged and elderly: a longitudinal study

**DOI:** 10.1186/s12199-020-00882-5

**Published:** 2020-09-06

**Authors:** Qing Wang, Jose A. Tapia Granados

**Affiliations:** 1 Department of Biostatistics, School of Public Health, Cheeloo College of Medicine, Shandong University, Jinan, 250100 Shandong China; 2 Institute for Medical Dataology, Cheeloo College of Medicine, Shandong University, Jinan, 250002 Shandong China; 3 Pudong Institute for Health Development, Shanghai, 200122 China; 4 College of Arts & Sciences, Drexel University, Philadelphia, PA USA

**Keywords:** Household heating, Indoor air pollution, Activities of daily living, Instrument activities of daily living, Chinese

## Abstract

**Background:**

The health hazards of indoor air pollution are well-established but studies of the health effects due to pollution from heating are rare. This study investigated the association of heating and disability for activities of daily living among Chinese middle-aged and elderly.

**Methods:**

We used two consecutive surveys in a cohort of over 17,000 adults aged 45 or older, who were interviewed first in 2011–2012 and then in 2013. In these surveys, taking advantage of random survey time, we applied a random effects logit regression model that included an interaction between pollution-producing heating fuel and a dummy variable, which measured interview time based on whether or not it was heating season.

**Results:**

Exposure to pollution-producing heating fuel was associated with a 39.9% (OR 1.399; 95%CI 1.227–1.594) and 71.0% (OR 1.710; 95%CI 1.523–1.920) increase in the likelihood of disability in activities of daily living (DADL) and disability in instrumental activities of daily living (DIADL), respectively. In heating season between year 2011 and 2013, moving from clean heating energy for heating to pollution-producing fuel was linked with an increase in the likelihoods having DADL and DIADL, with the OR of 2.014 (95%CI 1.126–3.600) and 1.956 (95%CI 1.186–3.226), respectively. However, disability increases due to change from clean energy to pollution-producing heating energy did not appear in advantaged education respondents.

**Conclusions:**

We found that exposure to heating by burning of coal, wood, or crop residue was associated with disability in performing daily living activities. Health policymakers should take indoor pollution due to heating into consideration as it is a major determinant of activities of daily living in elderly people; especially, such policy should focus on elderly people who have disadvantaged education.

## Introduction

According to the World Health Organization (WHO), almost half of the world population continues to depend on polluting fuels, including biomass fuels (wood, dung, and agricultural residues) and coal, for their energy needs [1]. WHO has estimated that 7.7% of global mortality is due to exposure to indoor air pollution from energy consumption, with 3.8 million deaths due to this cause in 2016 [2]. However, these numbers could be substantially underestimated since WHO uses estimates of the populations employing solid fuels for cooking as a proxy for indoor air pollution and does not take indoor air pollution from heating into account [3, 4].

Energy consumption is a major driving force of air pollution in many Asian developing countries [5]. Unlike other areas relying on solid fuels, including most regions of India and sub-Saharan Africa where heating needs are rare, in China, a substantial amount of solid fuels are used for heating [6]. China is an ideal example to estimate the health effects of indoor air pollution due to heating, considering the large variability of demand for heating from the north to the south of the country and a large solid-fuel-dependent population [5]. The heating period in cold areas (northeast and west) can last for as long as 200 days per year, while in other areas, it might be around 90 days [7]. According to a nationwide residential energy consumption survey, heating accounts for almost 50% of total residential energy use in China [8]. For households without access to central heating system, burning solid fuels in stoves is the most common home-heating way, known for high emissions of various pollutants and smoke backflow even for the improved types of stove [9–11].

Most previous studies have focused on cooking, and only a few studies have examined the health effects of residential heating in the context of China [12, 13]. Lin and Liu observed that indoor air pollution from coal burning significantly increased the diastolic and systolic blood pressure levels and likelihood of underweight [14]. Chen et al. estimated that premature deaths and disability-adjusted life years due to indoor air pollution exposure from heating in rural China were approximately 30,000 and 600,000 respectively per year in 2010 [15]. Since 2015, policies for substituting residential coal use by electricity have been implemented in the Beijing–Tianjin–Hebei region. Zhang et al. figured out that the health benefits would appear almost immediately after the individuals are no longer exposed to indoor air pollution from coal use [13]. Xue also designed a difference-in-difference framework that takes advantage of spatial and time variations in the implementing China’s centralized heating program; it found that the areas with centralized heating had a 1.2% decrease in the proportion of low-birth-weight infants [16].

In this study, using nationally wide individual-level data, we assessed the association between indoor air pollution due to heating and health among the middle elderly population, focusing on disability in activities of daily living (DADL) and disability in instrumental activities of daily living (DIADL). These indices quantify the disability to engage in those activities, which are essential for an independent life [16–18]. Disability is affecting millions of households and placing a burden on elderly people, care providers, and the care system [17–20]. Due to degrading environmental quality, there has been a rising interest in environmental determinants of activities of daily living (ADL) [18, 19]. However, to our knowledge, there is no one to explore the association of indoor air pollution from heating and ADL. The extended indoor staying time during the cold season makes exposure to household heating pollutants for the elderly an important concern for ADL in China. We expanded the analyses on the determinants of ADL to the influence of heating exposure. Furthermore, in order to identify socioeconomic disparities in heating-related ADL and figure out the vulnerable population, our analysis was stratified by education, which is a well-known measurement of socioeconomic status. The evaluation of DADL/DIADL due to heating might be useful for identifying potential interventions to prevent and reduce the health hazards.

## Methods

### Data

Data source was the 2011 and 2013 surveys of the China Health and Retirement Longitudinal Study (CHARLS). Basically, CHARLS, modeled after the US Health and Retirement Study, is a national representative sample of adults aged 45 or older, intended to investigate demographic issues, family structures, work status, retirement and pension conditions, income, various health-related variables, etc. [21]. CHARLS data were collected using stratified four-stage cluster sampling. In the first stage, 150 county-level units (rural counties and urban districts) were randomly selected from a sampling frame containing all county-level units of mainland China (excluding Tibet) in proportion to their population sizes (PPS). Within each county-level unit, 3 primary sampling units (PSUs)—administrative villages in rural areas or neighborhoods in urban areas—were randomly selected also following a PPS procedure. For sample cities, see Appendix figure 1. Within each PSU, 24 households with residents aged 45 years or older were randomly selected. Each resident aged 45 years or older and his or her spouse, if present, were interviewed face-to-face in each household. The general features of CHARLS have been detailed elsewhere [22]. This survey was approved by the ethics committee of the Institutional Review Board of Peking University (NO. IRB00001052-11014), and informed written consents were obtained from the participants.

The baseline survey was conducted between June 2011 and March 2012; it had an 80.51% response rate. Finally, the survey covered a total of 17,708 respondents. Among them, 15,196 respondents were followed up in a second wave of surveys conducted between July and December 2013, with a follow-up response rate of 88.30% . In total, 13,276 respondents answered the question on the main source of energy for household heating in both waves, which were used as our research sample. After excluding individuals younger than 45 years old and those with missing values in demographic, educational, health behaviors, and number of chronic diseases variables, the final sample size was 12,994 and 13,092 in 2011 and 2013, respectively.

In order to identify heating season at samples cities, the daily meteorological data at city-level was collected from the China Meteorological Data Sharing Service System—China Ground Climate Daily Data.

### Measures

#### Heating fuel

Each respondent was asked “Does your residence have central heating?” If the respondent said no, he or she was asked about the main heating energy source, including solar, coal, natural gas, liquefied petroleum gas, electric, crop residue/wood burning, and other. If the respondent used central heating, solar, natural gas, liquefied petroleum gas, electric as main source of energy, the dummy “pollution-producing heating fuel” was 0; the dummy was 1 as crop residue, wood, or coal were used as heating fuel. For those reporting heating fuel in their residence as “other” in 2011 and 2013, the majority (70.4%) of them lived in tropical and subtropical zones, so they probably do not need fuel for heating and were presented as 2.

Heating season is defined as the days when daily average outdoor temperature is steadily less than or equal to 5 °C, namely, all the 5-day running mean temperatures are less than or equal to 5 °C during the heating season [23, 24]. Following Liu et al. [25], the heating season in each sample city was identified. For respondents interviewed during the heating season, the dummy variable of “heating season” was encoded as 1, and 0 otherwise.

#### DIAD and DADL

DIADL scores quantify the ability to engage in 5 tasks implying making decisions and interacting with the environment, including (1) shopping, (2) managing own money, (3) cooking, (4) housekeeping, and (5) taking medicines [26]. DADL scores quantify the disability for performing 6 activities: (1) bathing, (2) dressing, (3) eating, (4) transferring from bed, (5) walking, and (6) toileting [26]. In assessing DIADL and DADL, each question on these activities can be answered “No, I don’t have any difficulty” (1 point); “I have difficulty but can still do it” (2 points); “Yes, I have difficulty and need help” (3 points), or “I cannot do it” (4 points). Thus, the total score of DADL can vary from 6 to 24 while the DIADL score from 5 to 20. Respondents with a DADL score higher than 6 or a DIADL score higher than 5 had difficulty in at least one activity. In these cases, the dummy variables of “DADL/DIADL” were encoded as 1, and 0 otherwise.

### Statistical analysis

The association between heating energy and DIADL/DADL was estimated with a random effects (RE) logistic model, which included an interaction between heating energy and the heating season. A modeling issue in longitudinal datasets is the heterogeneity among any sub-groups. In order to solve the issue, stratified estimation or a dummy variable must be inserted to account for the subsistence of the different sub-group. However, the fixed or random effects models are generally preferred for their simplicity in handling and normalizing heterogeneity in the error term. The random effect model (juxtaposed to a fixed effect model) has been opted for this study, because the individual in our sample is perceived as a random variable being part of a larger population. While in fixed models, the interest lays in the individual means across the levels of the fixed factor, in random effect models, the interest lays in the variance of means across the levels of a random factor. The selection of the random effect model is further strengthened after passing two tests (Breush–Pagan LM, and Hausman chi-square). The results of the two tests were not included in the manuscript, which could be obtained by contacting the authors. The RE logit model equation is presented as follows:

$$ P\left({D}_{ijt}=1\right)={b}_0+{e}_{jt}{b}_1+{w}_{ijt}{b}_2+{\mathrm{e}}_{\mathrm{jt}}\times {w}_{ijt}{b}_3+{X}_{ijt}{b}_4+{\mathrm{u}}_{\mathrm{i}}+{\mathrm{v}}_{\mathrm{t}} $$

where *D*_*ijt*_ *= 1* indicates person *i* in city area *j* in year *t* having either DADL or DIADL; *b*_0_, *b*_1_, *b*_2_, *b*_3_, and *b*_4_ are the parameters to be estimated; and *u*_*i*_ and *v*_*t*_ are RE dummy variables for individuals and the waves of CHARLS (CHARLS 2011, CHARLS 2013), respectively. All time-invariant factors that influence DADL/DIADL, such as family medical history and culture, are accounted for by the individual-specific constant terms *u*_*i*_, while abrupt events are captured by the survey year dummy variable *v*_*t*_; and *e*_*jt*_ is a dummy variable for pollution-producing heating fuel when the interview had done; *w*_*ijt*_ is a dummy variable that measured whether the respondents were interviewed during the heating season (dummy equal 1) or not (dummy equal 0). Furthermore, *e*_*jt*_ *× w*_*ijt*_ is the interaction between the dummy variable for the individual interview occurring during the heating season and using the household heating energy source at the interview time. Since CHARLS interview time is an exogenous variable for each respondent, we could compare heating-related-variation in DADL/DIADL  induced by this exogenous event through the estimated interaction parameters, which would be closer to the association between heating and ADL. More specifically, the estimated OR of interaction between the dummy variable for the interview occurring during the heating season and using the household heating energy source indicates [*P*(*D*_*ijt*_ = 1*|X*_*ijt*_, *w*_*ijt*_ = 1, *e*_*jt*_ = 1) − *P*(*D*_*ijt*_ = *1|X*_*ijt,*_ *w*_*ijt*_ = 1, *e*_*jt*_ = 0)] − [*P*(*D*_*ijt*_ = 1*|X*_*ijt*_, *w*_*ijt*_ = 0, *e*_*j*_ = 1) − *P(D*_*ijt*_ = 1*|X*_*ijt*_, *w*_*ijt*_ = 0, *e*_*jt*_ = 0)]. These defined variables can be interpreted as the variations in DADL or DIADL, respectively, due to using pollution-producing fuel for heating, excluding other factors. [*P*(*D*_*ijt*_ = 1*|X*_*ijt*_, *w*_*ijt*_ *=* 1, *e*_*jt*_ *=* 1) − *P(D*_*ijt*_ = 1*|X*_*ijt*_, *w*_*ijt*_ = 1, *e*_*jt*_ = 0)] reflects variations in DADL or DIADL due to the shift of clean heating fuel to pollution-producing fuel during the heating season, while [*P*(*D*_*ijt*_ = 1*|X*_*ijt*_, *w*_*ijt*_ = 0, *e*_*j*_ = 1) − *P*(*D*_*ijt*_ = 1*|X*_*ijt*_, *w*_*ijt*_ = 0, *e*_*jt*_ = 0)] is variations in DADL or DIADL using different heating energies in the nonheating season. We assumed that both DADL and DIADL among respondents with different survey times are comparable. This interaction is documented in variations in DADL and DIADL due to pollution-producing heating when other influencing factors could be captured using [*P*(*D*_*ijt*_ = 1*|X*_*ijt*_, *w*_*ijt*_ = 0, *e*_*j*_ = 1) − *P*(*D*_*ijt*_ = 1*|X*_*ijt*_, *w*_*ijt*_ = 0, *e*_*jt*_ = 0)]. Therefore, the estimated OR of interaction term may allow identification of the association between the heating energy source and DIADL or DADL. In addition, the variations in DADL/DIADL within 2 years were observed, helping us to figure out if the heating was an acute influencing factor.

*X*_*ijt*_ represents the vector of determinants of DIADL or DADL, including demographic characteristics, socioeconomic status, health behaviors, and health status stated in a conceptual framework of WHO [27]. Demographic characteristics included age, sex, and marital status (common-law marriage was considered as married). Education, indicating socioeconomic status, was defined as disadvantage (primary school or below) and advantage group (junior high school or above).

Smoking status (current smoker vs non-smoker with non-smoker as the reference group) and alcohol drinking status (heavy drinker vs non-heavy-drinker) were also included as indicators of health behaviors. Respondents were asked if they drank beer or any other alcoholic beverage during the previous 12 months. Those who responded in the affirmative were asked further questions on the type of beverage (beer, grape wine, liquor) and the typical amount of alcoholic drinks they consumed on a single occasion (reported by the number of bottles (640 ml) for beer and number of liang (50 g) for wines and liquor), assuming the following alcohol content by volume (v/v) to be typical in China, beer 4%, grape wine 12%, and liquor 45%. Heavy drinking episodes were classified as the consumption of more than 60 g of alcohol on one occasion for men, and more than 40 g for women [28].

The number of chronic diseases was used to measure physical health. Respondents were asked if a physician diagnosed that they had any chronic disease. If the answer was “yes,” the respondent was asked further questions about the name of the chronic disease. The number of chronic diseases was categorized into 3 groups: 0, 1, and 2 or more above.

We controlled the main source of cooking fuel in order to isolate the effects of heating. For those who used coal or crop residue/wood burning as the main energy source, the dummy “pollution-producing cooking fuel” was 1, and for all others, it was 0. Finally, dwelling conditions were adjusted in the regression estimation. If a house was over 15 years old and the type of the house structure type was neither concrete and steel nor bricks and wood, the housing condition was encoded as unfavorable.

Separate regressions were computed using the observations in the group with primary school and below or junior high school and above group. Education is a well-recognized measurement of socioeconomic status, and respondents with higher education usually could afford to pay for more expensive fuel (e.g., coal with less sulfur) or central heating system so that pollution is to a greater or lower extent avoided. In addition, there is evidence of education as an indicator of health knowledge [29]. Odds ratio (OR) and 95% confidence interval (CI) were reported in all the regressions. STATA 14 was used for all calculations.

## Results

Table 1 summarizes the characteristics of the participants in the study. The average age of the respondents at the first interview (June 2011 to March 2012) in the current study were 59.4 (SD 9.6), with 51.6% females. Among them, 12.4% were unmarried. Only 32.4% of the respondents had finished high school. Coal or wood was used respectively by 56.5 and 56. 4% of the interviewed residents as fuel for heating or cooking, and 21.0 and 19.0% of respondents reported having DIADL and DADL, respectively.

**Table 1:** Characteristics of the CHARLS participants

	2011		2013	
	Observations	Statistic^a^	Observations	Statistic
Age	12,994	59.4 (9.6)	13,092	61.3 (9.7)
Sex	12,994		13,092	
Female	6705	51.6%	6808	52.0%
Male	6289	48.4%	6284	48.0%
Marital status	12,994		13,092	
Unmarried	1611	12.4%	1794	13.7%
Married	11,383	87.6%	11,298	86.3%
Education level	12,994		13,092	
Junior high sch. or more	4210	32.4%	4242	32.4%
Primary sch. or below	8784	67.6%	8850	67.6%
Smoking status	12,994		13,092	
Current smoker	4132	31.8%	4870	37.2%
Non-smoker	8862	68.2%	8222	62.8%
Alcohol drinking status	12,994		13,092	
Heavy drinker	923	7.1%	1165	8.9%
Non-heavy-drinker	12,071	92.9%	11,927	91.1%
Cooking fuel	12,994		13,092	
Pollution-producing cooking fuel	7329	56.4%	6,310	48.2%
Others	5665	43.6%	6782	51.8%
Housing condition	12,994		13,092	
Unfavorable housing condition	1377	10.6%	1100	8.4%
Favorable housing condition	11,616	89.4%	11,992	91.6%
Number of chronic disease	12,994		13,092	
None	4210	32.4%	5263	40.2%
One	3872	29.8%	3980	30.4%
Two or more	4899	37.7%	3849	29.4%
Heating fuel	12,994		13,092	
Clean fuel	3469	26.7%	4242	32.4%
Pollution-producing heating fuel	7341	56.5%	6886	52.6%
Other	2183	16.8%	2016	15.4%
Surveyed in heating season	725	5.6%	312	2.4%
DIADL	12,905		13,092	
Yes	2710	21.0%	2749	21.0%
No	10,195	79.0%	10,343	79.0%
DADL	7764		13,092	
Yes	1475	19.0%	3325	25.4%
No	6289	81.0%	10,002	74.6%

*DIADL* disability for instrumental activities of daily living, *DADL* disability for activities of daily living

^a^Statistics are means with bracketed standard deviations; or percentage when thus indicated

In 2013, the mean age was higher, and a higher percentage of respondents reported poor health behaviors and health status. However, using clean fuel for heating and cooking were more prevalent. Thirty-two respondents had been interviewed twice in the heating season; 280 in the heating season of 2013 and not in the heating season of 2011; and 693 in the heating season of 2011 and not in the heating season of 2013. From 2011 to 2013, about 4.9% (650) of the respondents moved from clean to pollution-producing heating fuel, while 9.9% (1318) moved from pollution-producing to clean heating fuel (Appendix figure 2). A higher prevalence of heating uses solid fuels in warm, medium, or cold temperature zones and in plateau-climate zones where heating season is longer, but wood or crop residues and coal were widely used in all temperature zones (Appendix table 1). Compared to the more highly educated respondents, those with lower educational attainment who also used pollution-producing heating fuel were more prevalent. In 2011, the proportion of households burning coal, wood, or crop residue for heating in low-education respondents was 61.6% but only 45.9% in high-education respondents. Similar proportions were observed in 2013 (Appendix table 2).

Table 2 shows associations between the heating energy source and either DADL or DIADL. The OR of heating fuel suggested exposure to coal or wood burning for heating was positively related to either DADL or DIADL for Chinese middle-aged and elderly. Using pollution-producing fuel for heating was associated with a 39.9% (OR 1.399; 95%CI 1.227–1.594) and 71.0% (OR 1.710; 95%CI 1.523–1.920) increase in the likelihood of DADL and DIADL.

**Table 2:** Multiple regression analysis of associations between household heating and disability for activities of daily living in random effects logit models

	Model 1 DADL^a^	Model 2 DIADL^b^
	OR^c^ (95%CI)	OR^c^ (95%CI)
Heating season	0.753	0.763
	(0.470–1.205)	(0.512–1.138)
Heating fuel		
Pollution-producing heating fuel	1.399***^d^ (1.227–1.594)	1.710*** (1.523–1.920)
Other heating fuel	1.040 (0.885–1.223)	1.155** (1.002–1.331)
Interaction of pollution-producing heating fuel and heating season	2.014** (1.126–3.600)	1.956*** (1.186–3.226)
Interaction of other heating fuel and heating season	0.357*** (0.167–0.764)	0.501** (0.271–0.924)
Age	1.051*** (1.044–1.057)	1.062*** (1.056–1.068)
Female	1.050 (0.923–1.195)	1.515*** (1.349–1.702)
Unmarried	1.238*** (1.076–1.425)	1.279*** (1.123–1.458)
Junior high sch. or more	0.729*** (0.640–0.832)	0.516*** (0.459–0.580)
Heavy drinker	0.850 (0.681–1.062)	0.825** (0.688–0.989)
Current smoker	0.865** (0.758–0.987)	0.887** (0.789–0.997)
Pollution-cooking cooking fuel	1.327*** (1.186–1.483)	1.452*** (1.316–1.601)
Unfavorable housing conditions	1.362*** (1.171–1.584)	1.542*** (1.348–1.764)
Number of chronic disease		
One	1.721*** (1.502–1.972)	1.503*** (1.339–1.688)
Two or more	3.050*** (2.673–3.480)	3.184*** (2.845–3.564)
Wave 2013	1.622*** (1.480–1.778)	1.039 (0.963–1.120)
Observations	16,634	25,156

^a^*DADL* disability for activities of daily living. Model 1 presents the association between heating fuel and DADL

^b^*DIADL* disability for instrumental activities of daily living. Model 2 presents the association between heating fuel and DIADL

^c^95%CI (confidence interval) bracketed below the odds ratio (OR) estimates

^d^*p* < 0.1

**p < 0.05

***p < 0.01

Insignificant differences in DADL/DIADL and demographic characteristics were observed between respondents surveyed in heating season and non-heating season (Appendix table 3). Estimating the interaction between heating energy and individual interview time during the heating season shows that moving from clean heating energy to pollution-producing fuel was  linked with an increase in the likelihoods having DADL and DIADL in heating season between year 2011 and 2013, with ORs of 2.014 (95%CI 1.126–3.600) and 1.956 (95%CI 1.186–3.226), respectively. Since education was significantly associated with increases in DADL and DIADL, we further stratified our estimates by education.

Table 3 shows associations of the source of energy for heating and either DADL or DIADL by education. Pollution-producing heating fuel was associated with increases in both DADL and DIADL in estimates stratified by education. However, judging from the interaction between interview time and heating fuel, in the group who had a junior high school education or higher, there were no significant differences in DADL/DIADL likelihoods due to changes of heating fuel in heating season during the period from year 2011 to 2013, whereas, for respondents with a primary school education or lower, changes in heating energy from clean energy to coal, or wood/crop residue was related to an increased OR in DADL (OR 2.428; 95%CI1.191–4.953) and DIADL(OR 2.234; 95%CI 1.199–4.163).

**Table 3:** Multiple regression analysis of associations between household heating and disability for activities of daily living by education in random effects logit models

	Primary school and below		Junior high sch. or more	
	Model 1 DADL^a^	Model 2 DIADL^b^	Model 3 DADL^a^	Model 4 DIADL^b^
	OR^c^ (95%CI)	OR^c^ (95%CI)	OR^c^ (95%CI)	OR^c^ (95%CI)
Heating season	0.664 (0.357–1.235)	0.642 (0.376–1.097)	0.900 (0.411–1.972)	0.980 (0.527–1.821)
Heating fuel				
Pollution-producing heating fuel	1.285***^d^ (1.109–1.488)	1.580*** (1.384–1.804)	1.894*** (1.405–2.552)	2.119*** (1.661–2.703)
Other heating fuel	0.980 (0.820–1.171)	1.127 (0.962–1.321)	1.219 (0.818–1.817)	1.087 (0.779–1.518)
Interaction of pollution-producing heating fuel and heating season	2.428** (1.191–4.953)	2.234** (1.199–4.163)	0.845 (0.191–3.751)	2.196 (0.736–6.546)
Interaction of other heating fuel and heating season	0.336*** (0.152–0.743)	0.531* (0.279–1.009)	0.675 (0.061–7.431)	0.276 (0.039–1.956)
Age	1.049*** (1.042–1.056)	1.062*** (1.056–1.068)	1.058*** (1.042–1.074)	1.059*** (1.046–1.073)
Female	1.089 (0.945–1.254)	1.633*** (1.433–1.861)	0.916 (0.668–1.258)	1.172 (0.906–1.515)
Unmarried	1.200** (1.037–1.388)	1.279*** (1.114–1.468)	1.640** (1.046–2.569)	1.168 (0.792–1.723)
Heavy drinker	0.795* (0.607–1.042)	0.866 (0.693–1.082)	0.990 (0.653–1.500)	0.723* (0.522–1.002)
Current smoker	0.910 (0.787–1.053)	0.926 (0.810–1.058)	0.719** (0.528–0.979)	0.779** (0.610–0.994)
Pollution-cooking cooking fuel	1.342*** (1.188–1.515)	1.415*** (1.270–1.578)	1.268* (0.960–1.674)	1.545*** (1.234–1.935)
Unfavorable housing conditions	1.368*** (1.168–1.602)	1.560*** (1.351–1.801)	1.312 (0.838–2.053)	1.432** (1.000–2.049)
Number of chronic disease				
One	1.719*** (1.482–1.994)	1.430*** (1.258–1.625)	1.729*** (1.232–2.427)	1.825*** (1.397–2.384)
Two or more	2.957*** (2.561–3.413)	2.881*** (2.542–3.264)	3.505*** (2.520–4.877)	4.622*** (3.571–5.982)
Wave 2013	1.580*** (1.429–1.748)	1.018 (0.935–1.107)	1.864*** (1.492–2.328)	1.115 (0.940–1.323)
Observations	12,199	16,953	4428	8191

^a^*DADL* disability for activities of daily living. Models 1 and 3 present the association between heating fuel and DADL for respondents with primary school or below and junior high school or above, respectively

^b^*DIADL* disability for instrumental activities of daily living. Models 2 and 4 present the association between heating fuel and DIADL for respondents with primary school or below and junior high school or above, respectively

^c^95%CI (confidence interval) bracketed below odds ratio (OR) estimates

^d^*p < 0.1

**p < 0.05

***p < 0.01

We also observed that the association between pollution-producing cooking and DIADL or DADL were positive and significant, which aligns with our views that household pollution may negatively affect ADL. In addition, unfavorable housing conditions and the number of chronic diseases were also related with worsen DADL and DIADL.

Age is a strong factor of both DADL and DIADL, with an extra year of life was linked, on average, with approximately a 5–6% increase in the likelihood of DADL or DIADL (Tables 2 and 3).

We found a negative association between unhealthy behaviors (e.g., alcohol drinking, smoking) and DIADL/DADL. The association may exist because those with DADL or DIADL may have ceased smoking or heavy drinking due to their DADL/DIADL status, so we observed a negative association between unhealthy behaviors and DIADL/DADL.

## Discussion

Exploiting available nationally representative, individual data on social-demographic characteristics and health, we investigated household heating in China’s middle-aged and elderly population. We found health hazards that stemmed from exposure to pollution-producing heating. During the year 2011 and 2013, variations in DADL or DIAD were associated with changes from clean energy to coal and biomass fuels for heating.

With liquid and gaseous fuels, it is easy to mix properly fuel and air during burning, but this is not the case with solid fuels. Solid fuels (biomass and coal) are difficult to burn fully in simple combustion devices such as household heating stoves and the incomplete combustion produces substantial emissions of pollutants such as carbon monoxide (CO), nitrogen dioxide (NO_2_), and particulate matter [30–32]. In households that use sulfur-rich coal, reported concentrations of sulfur dioxide (SO_2_) also often exceed China’s indoor air quality standards [31].

Because both CO and NO_2_ can penetrate a person’s bloodstream, these chemicals can affect the central nervous system and alter its functions. Furthermore, both CO and NO_2_ and particulate matter have a local effect on the respiratory airways, interfering with the capture of the O_2_ required by the organism and the elimination of CO_2_ produced by the metabolism [33, 34]. This information can explain not only cognitive and neurological disturbances developing from household air pollution but also other harmful effects on a person’s health, including weakening of the immune system and reducing lung function, which resulted in increased mortality and hospital admissions due to respiratory and cardiovascular disease [33–35]. In addition, particulate matter may enter by direct translocation through the olfactory bulb and then could elicit inflammatory responses and oxidative stress, causing respiratory or cardiac symptoms, as well as chronically, potentially affecting every organ in the body [33, 36, 37]. As adverse outcomes of frailty, disability is often accompanied by the development of the aforementioned chronic diseases [33, 37–39]. Individuals with multidiseases at later and more severe stages and who have less access to therapeutic interventions tend to report higher likelihood of DADL/DIADL [39].

Results from different models show the biologically plausible effect of aging on disability, since an extra year of life was associated with a 5–6% average increase in ORs of DADL or DIADL. Comparing this estimate with pollution-producing heating estimate can help quantify its importance. Judging from the ORs and their 95% CIs which have very little overlap, the effect size of indoor pollution due to burning of pollution-producing fuels was obviously greater for DADL/DIADL than that of aging.

We also note that, compared to respondents who have a junior high school or above, respondents who have a primary school education or lower had more exposure to heating by burning of coal, wood, or crop residue. Futhermore, for those with disadvantaged education, variation in the likelihood of DADL/DIADL was found due to changing between clean energy and pollution-producing fuel in heating season between year 2011 and 2013. However, such an association did not exist for respondents with advantaged education. The hypothesis is that the highly educated individuals may find it much easier to contain health risk from indoor pollution. As a factor influencing health knowledge and socioeconomic status, education could improve respondents’ awareness and ability (e.g., investment toward environmental infrastructure inside the household) to protect from indoor air pollution due to heating, so that those with disadvantage education were more vulnerable to harmful health effects due to pollution-producing heating fuel [37].

China is a global leader in renewable energy. China’s 12th Five-Year Plan identified renewables as an emerging strategic industry and established targets for thermal applications of renewable energy sources [7]. However, health risks related to indoor air pollution from heating have received much less attention. To our knowledge, this paper for the first time documents in a national sample from China the associations of heating by using pollution-producing fuels with DIADL and DADL.

Policymakers should consider the evidence of the harmful aspects of indoor pollution due to heating, and interventions aimed at reducing DADL/DIADL should be strengthened in the heating season. Even in the tropical and subtropical zone, the proportion to use heating equipment for warm purposes is relatively high among the elderly. Therefore, the issue in the tropical and subtropical zone should be taken into account, in particular, the lack of pollutant reduction control and regulation in these areas [38].

Our comparison between individuals pertaining to different levels of education highlights the heterogeneous impacts of heating within different strata of the same population who share life habits and cultural customs, thus avoiding confounding factors that are present in cross-country studies. In this case, our investigation identified the vulnerable population in which the reduction of the health hazard from pollution-producing fuel for heating is a priority. Since households with disadvantaged education were more vulnerable to the adverse consequences of heating, health promotion policies including the development of centralized home heating infrastructure should be specifically focused on these households.

The limitations of the study are also of note. First of all, we only provide indirect evidence for the association between pollution-producing heating fuel use and DADL/DIADL. The explanations of biological plausibility and mechanisms are more restrained—no exposure measurements are made here. Secondly, we did not insufficiently adjust control variables including outdoor air pollution, second-hand smoking, and other air pollutants, which may comprise a limitation in this study due to potential confounding [37]. For example, unfavorable heating conditions may promote increased outdoor activity, such as sitting in the sun, which is popular for older Chinese people. Simultaneously, outdoor air pollution levels in winter are among the worst in China, since a large portion of air pollutants result from residential combustion of solid biomass and coal fuel for heating. This exposure to additional outdoor air pollutants may also increase disability. In addition, influencing factors of ADL such as family medical history were not adjusted due to data limitation.

Another source of bias can be that the survey or our interpretation of it did not allow for multiple heating fuels (i.e., heating with clean energy sources and coal/wood). And a short period of follow-up which implies restricted variability of the observations. All of these emphasize the need of confirmatory studies that may enhance the generalizability of our findings. However, the variation in DADL/DIADL occurred within 2 years (from year 2011 to 2013), suggesting the heating was an acute influencing factor.

## Conclusion

DADL or DIADL was associated with exposure to coal and biomass fuels for heating. During year 2011 and 2013, the variation in either DADL or DIADL was observed as clean heating energy changed to pollution-producing energy in the heating season. However, this variation in DADL/DIADL due to changes in heating energy did not appear in more highly educated respondents. Healthcare policymakers should take indoor pollution due to heating into consideration, as it is a major determinant of DADL in the elderly population, and focus resulting policies on elderly people with disadvantages in education.

## Supplementary information

**Additional file 1:** **Appendix Fig.1.** Sample cities. **Appendix Fig.2.** Observation of observations by source of energy for household heating from wave 2011 to 2013. **Appendix Table 1.** Percentage of households by source of energy for household heating (%). **Appendix Table 2.** Percentage of households by source of energy for household heating in education group (%). **Appendix Table 3.** Demographic statistics by respondents surveyed in heating season and non-heating season.

## Data Availability

CHARLS is a public open-access dataset. Down link: http://charls.pku.edu.cn/en.

## Abbreviations

WHO: World Health Organization
PPS: Proportion to their population sizes
PSUs: Primary sampling units
ADL: Activities of daily living
DADL: Disability in activities of daily living
DIADL: Disability in instrumental activities of daily living
