# Supplementary material for: Household heating associated with disability in activities of daily living among Chinese middle-aged and elderly: a longitudinal study
Source: Environ Health Prev Med. 2020 Sep 6;25:49. doi: 10.1186/s12199-020-00882-5 (PMC7487906; doi:10.1186/s12199-020-00882-5)
Supplement: Supplementary file 1 — Additional file 1: Appendix Fig.1. Sample cities. Appendix Fig.2. Observation of observations by source of energy for household heating from wave 2011 to 2013. Appendix Table 1. Percentage of households by source of energy for household heating (%). Appendix Table 2. Percentage of households by source of energy for household heating in education group (%). Appendix Table 3. Demographic statistics by respondents surveyed in heating season and non-heating season. [file 12199_2020_882_MOESM1_ESM.docx]

**Appendix Fig.1** Sample cities


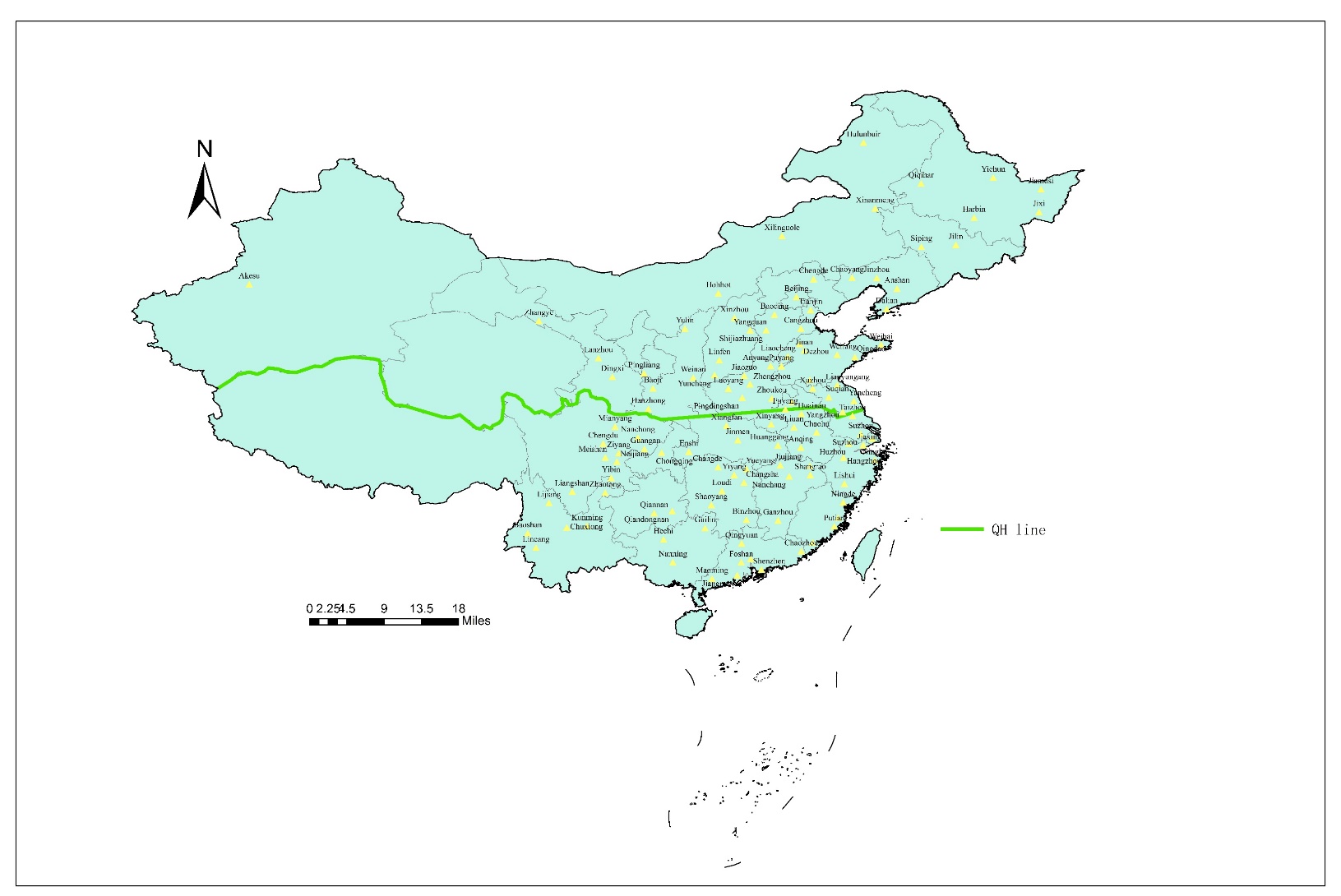


QH line: Qinling Mountains-Huaihe River line, which divides into tropical/subtropical temperature zone and warm, media, cold temperature and plateau climate zone

**Appendix Fig.2** Observation of observations by source of energy for household heating from wave 2011 to 2013

| 2013 2011 | Central heating | Solar | Natural gas | Liquefied Petroleum Gas | Electric | Coal | Crop residue/Wood | Other |
| --- | --- | --- | --- | --- | --- | --- | --- | --- |
| Central heating | 773 | 14 | 16 | 14 | 56 | 441 | 105 | 45 |
| Solar | 0 | 9 | 3 | 2 | 61 | 21 | 30 | 58 |
| Natural gas | 21 | 8 | 39 | 15 | 39 | 30 | 32 | 40 |
| Liquefied Petroleum Gas | 4 | 0 | 8 | 7 | 17 | 17 | 13 | 39 |
| Electric | 88 | 85 | 69 | 71 | 800 | 284 | 345 | 548 |
| Coal | 163 | 25 | 15 | 36 | 153 | 2365 | 552 | 125 |
| Crop residue/Wood | 27 | 24 | 12 | 14 | 181 | 354 | 2316 | 606 |
| Other | 36 | 61 | 60 | 70 | 436 | 164 | 429 | 785 |

**Appendix Table 1**. Percentage of households by source of energy for household heating (%)

|  | 2011 heating energy source | |  |
| --- | --- | --- | --- |
|  | Tropical and Subtropical zone  (N=6,038) | Warm, medium and cold temperature zone  /plateau climate zone (N=7,238) |  |
| Heating system | | 1.6 | 14.4 |
| Solar | | 2.5 | 1.1 |
| Natural gas | | 2.9 | 0.6 |
| Liquefied Petroleum Gas | | 2.6 | 1.0 |
| Electric | | 21.1 | 6.5 |
| Coal | | 11.7 | 41.0 |
| Wood/crop residue | | 34.4 | 24.1 |
| Other | | 23.2 | 11.7 |
|  | |  |  |
|  | | 2013 heating energy source | |
|  | | Tropical and Subtropical zone  (N=6,038) | Warm, medium and cold temperature zone  /plateau climate zone (N=7,238) |
| Heating system | | 1.7 | 18.8 |
| Solar | | 2.0 | 0.9 |
| Natural gas | | 2.6 | 0.9 |
| Liquefied Petroleum Gas | | 1.1 | 0.5 |
| Electric | | 27.1 | 9.1 |
| Coal | | 8.6 | 40.3 |
| Wood/crop residue | | 33.7 | 20.7 |
| Other | | 23.3 | 8.8 |

**Appendix Table 2.** Percentage of households by source of energy for household heating in education group (%)

|  | 2011 | | 2013 | |
| --- | --- | --- | --- | --- |
|  | Primary school and below | Junior high sch. or more | Primary school and below | Junior high sch. or more |
|  | N=8,981 | N=4,276 | N=8,981 | N=4,276 |
| Heating system | 4.3 | 16.9 | 7.1 | 19.3 |
| Solar | 1.6 | 1.9 | 1.4 | 1.3 |
| Natural gas | 1.3 | 2.4 | 1.6 | 2.0 |
| Liquefied Petroleum Gas | 1.4 | 2.5 | 0.9 | 0.6 |
| Electric | 11.2 | 17.2 | 15.8 | 20.4 |
| Coal | 26.7 | 29.8 | 25.0 | 27.7 |
| Wood/crop residue | 34.9 | 16.1 | 32.1 | 15.2 |
| Other | 16.2 | 13.1 | 16.2 | 13.7 |

**Appendix Table 3**. Demographic statistics by respondents surveyed in heating season and non-heating season

|  | Respondents surveyed in heating season (1,037) | Respondents surveyed in non-heating season(25,049) |
| --- | --- | --- |
| Junior high sch. or more | 33.3% | 32.3% |
| Age | 59.8(10.4) | 60.0(9.9) |
| Female | 53.5% | 52.5% |
| DIADL |  |  |
| Yes | 21.5% | 21.1% |
| No | 78.5% | 78.9% |
| DADL |  |  |
| Yes | 22.9% | 22.5% |
| No | 77.1% | 77.5% |

^a^ DIADL: disability for instrumental activities of daily living;

^b^ DADL: disability for activities of daily living.

^c^ Statistics are means with bracketed standard deviations; or percentage when thus indicated

^d^ Number of respondents reporting DADL and DIADL was 595 and 964 in heating season; number of respondents reporting DADL and DIADL was 20261 and 25,033 in non-heating season.
